# Hemp Seed-Derived Vesicle-like Nanoparticles Modulate Dermal Collagen Homeostasis and Attenuate UVB-Induced Photoaging-Related Responses

**DOI:** 10.3390/molecules31193455

**Published:** 2026-09-28

**Authors:** Hannah S. Park, Sehyun Shin

**Affiliations:** 1 Engineering Research Center for Biofluid Biopsy, Seoul 02841, Republic of Korea; 1nashin@korea.ac.kr; 2 School of Mechanical Engineering, Korea University, Seoul 02841, Republic of Korea

**Keywords:** hemp seed, vesicle-like nanoparticles, electrokinetic isolation, extracellular matrix homeostasis, photoaging, melanogenesis

## Abstract

Plant-derived extracellular vesicle-like nanoparticles (EVNPs) have emerged as bioactive nanomaterials with potential applications in skin biology; however, their isolation from complex plant matrices and their integrated biological effects remain insufficiently characterized. In this study, hemp seed-derived vesicle-like nanoparticles (hs- extracellular VLNPs) were isolated using a multi-step workflow combining pre-clarification, sequential filtration, electrokinetic capture, and tangential flow filtration. The isolated hs-VLNPs exhibited membrane-enclosed vesicle-like morphology, a nanoscale size distribution predominantly within 100–200 nm, and a negative surface charge. hs-VLNPs maintained cellular viability within the concentration range used for most functional assays and exhibited multiple biological activities associated with skin cellular homeostasis. In human dermal fibroblasts, hs-VLNPs increased procollagen type I production and attenuated UVB-induced matrix metalloproteinase-1 (MMP-1) expression following UVB exposure. In HaCaT keratinocytes, hs-VLNPs enhanced scratch closure under non-cytotoxic treatment conditions. Inflammatory mediator responses were cell- and mediator-dependent, with differential effects observed in RAW264.7 macrophages and human dermal fibroblasts rather than uniform suppression of inflammatory mediators. In addition, hs-VLNPs reduced α-melanocyte-stimulating hormone (α-MSH)-induced melanin production while exhibiting little direct inhibition of tyrosinase activity, suggesting that the anti-melanogenic effect may involve cellular regulatory mechanisms rather than direct enzymatic inhibition. Collectively, these findings demonstrate that electrokinetic capture combined with membrane-based filtration enables the enrichment of biologically active vesicle-like nanoparticles from hemp seeds. Rather than exerting a single biological effect, hs-VLNPs modulated multiple interconnected processes involving extracellular matrix homeostasis, cellular migration, immune responses, and melanogenesis, providing a foundation for further investigation of hemp seed-derived EVNPs as plant-derived nanomaterials for skin-related applications.

## 1. Introduction

Skin aging is a complex biological process driven by intrinsic factors and environmental stressors, among which ultraviolet (UV) radiation is considered one of the most critical contributors to photoaging. Ultraviolet B (UVB) irradiation induces the expression of matrix metalloproteinases (MMPs), particularly matrix metalloproteinase-1 (MMP-1), which degrades type I collagen in the dermal extracellular matrix [1,2,3].

The breakdown of collagen leads to structural deterioration of the dermis, resulting in wrinkle formation and loss of skin elasticity. Conversely, dermal fibroblasts continuously synthesize type I collagen to maintain skin structure and mechanical integrity. Therefore, maintaining collagen homeostasis, defined as the balance between collagen synthesis and degradation, is considered a key biological mechanism in preventing UV-induced skin aging [4,5].

Natural bioactive materials have long been explored as potential resources for skin regeneration and anti-aging applications. For example, *Centella asiatica* has been extensively studied for its wound healing and skin regenerative properties, and its active components such as madecassoside are known to stimulate collagen synthesis and promote dermal repair [6]. In addition, biologically derived materials from yeast, particularly *Saccharomyces cerevisiae*, have been widely utilized in cosmetic and biomedical applications due to their antioxidant and regenerative properties [7]. These studies highlight the potential of biological resources to modulate cellular functions relevant to skin repair and aging.

In recent years, extracellular vesicles (EVs) have emerged as important mediators of intercellular communication. EVs are nanoscale lipid bilayer vesicles that transport proteins, lipids, and nucleic acids, thereby regulating various physiological processes through cell-to-cell signaling [8]. Because of their ability to deliver bioactive molecules, EVs have attracted significant interest as potential therapeutic and cosmetic delivery platforms. Beyond mammalian systems, EV-like nanovesicles have also been identified in various plant species, and plant-derived EV-like nanoparticles (p-EVNPs) have recently been investigated as promising bioactive nanomaterials with anti-inflammatory, antioxidant, and tissue regenerative properties [9,10].

Independent studies using ginger-, *Morinda officinalis*-, and broccoli-derived nanoscale vesicular preparations have reported anti-inflammatory and tissue-reparative activities, while recent reviews highlight the diverse biological functions and expanding biomedical applications of plant-derived EVs and vesicle-like nanoparticles across a broad range of botanical sources [11,12,13,14,15].

*Cannabis sativa* is a biologically active plant species known to contain a wide range of bioactive compounds. Among its various tissues, hemp seed is particularly rich in unsaturated fatty acids, proteins, antioxidants, and other functional molecules, and has therefore been widely studied for nutritional and biomedical applications [16]. However, most previous studies have focused on hemp seed oil or solvent extracts, while nanoscale EV-like nanoparticles derived from hemp seed remain largely unexplored. In particular, seed tissues are characterized by high lipid content and complex cellular structures, which present technical challenges for the isolation of nanoscale vesicles.

Recent methodological guidance further cautions that vesicular preparations obtained following plant tissue disruption may contain heterogeneous membrane- or lipid-derived nanoparticles and other co-isolated particles, making extracellular origin difficult to establish without rigorous purification and characterization and supporting the use of conservative nomenclature when vesicle biogenesis has not been directly demonstrated [17,18,19].

In the present study, extracellular vesicle-like nanoparticles were isolated from hemp seeds and characterized for their physicochemical properties. These particles are herein referred to as hemp seed-derived vesicle-like nanoparticles (hs-VLNPs). The biological effects of hs-VLNPs were subsequently evaluated using multiple cellular models relevant to skin biology. Their effects on cell viability, inflammatory mediator responses, collagen homeostasis, wound healing, and melanogenesis-related processes were investigated to assess the potential of hs-VLNPs as a plant-derived nanomaterial for skin-related applications.

## 2. Results

To evaluate the biological effects of hemp seed-derived EV-like nanoparticles (EVNPs), EVNPs were first isolated using the electrokinetic filtration workflow illustrated in Figure 1. The isolated EVNPs were then characterized to confirm their structural and physicochemical properties prior to subsequent functional assays.

### 2.1. Isolation and Physicochemical Characterization of hs-VLNPs

The morphology of hemp seed-derived EV-like nanoparticles (hs-VLNPs) was examined using cryogenic transmission electron microscopy (cryo-TEM). Cryo-TEM imaging revealed spherical, membrane-enclosed nanostructures with clearly defined bilayer-like membranes, supporting their vesicle-like morphology (Figure 1b).

Nanoparticle tracking analysis (NTA) showed that the isolated hs-VLNP preparation contained approximately 1.7 × 10^10^ particles/mL, with a nanoscale size distribution predominantly within the range of approximately 100–200 nm, a mean particle diameter of 161.6 nm, and a median diameter (×50) of 145.7 nm (Figure 1c). These findings indicate that the isolation procedure yielded a population enriched in nanosized vesicle-like particles.

Surface charge characteristics were further assessed by zeta potential analysis. The hs-VLNPs exhibited a predominantly negative surface charge (Appendix A Figure A1). This physicochemical property is consistent with the electrokinetic capture principle of the ExoFilter system [20], in which negatively charged particles are preferentially retained through charge-mediated interactions with the positively charged capture matrix.

NTA showed that the isolated hs-VLNP preparation contained approximately 1.7 × 10^10^ particles/mL, with a mean particle diameter of 161.6 nm and a median diameter (×50) of 145.7 nm. The total protein concentration determined by BCA assay was 197 μg/mL, corresponding to a particle-to-protein ratio of approximately 8.63 × 10^7^ particles/μg protein (Appendix A Figure A1b). After approximately 6 months of storage, the hs-VLNP preparation showed no appreciable changes in particle size, vesicle-like morphology, or pH compared with the initial characterization under the tested storage conditions.

Collectively, the cryo-TEM, NTA, and zeta potential analyses demonstrated that the isolated hs-VLNPs possessed nanoscale dimensions, vesicle-like membrane morphology, and a negatively charged surface. These characteristics support the successful enrichment of EV-like nanoparticles using the integrated electrokinetic filtration workflow and provide a physicochemical basis for their subsequent biological and functional evaluation.

### 2.2. Cytotoxicity of hs-VLNPs in Cell Models Used for Functional Assays

Prior to the evaluation of biological activity, the cytotoxicity of hs-VLNPs was assessed in the cell models used for subsequent functional assays to establish appropriate treatment concentration ranges.

In HaCaT keratinocytes exposed to hs-VLNPs for 24 h, cell viability was 104.1 ± 2.8%, 105.09 ± 4.6%, 100.5 ± 3.4%, 95.4 ± 4.5%, and 76.8 ± 4.3% at concentrations of 2%, 5%, 10%, 20%, and 50% (*v*/*v*), respectively (Figure 2a). Thus, cell viability remained close to the control level at concentrations up to 20%, whereas a reduction was observed at 50%.

A similar concentration-dependent pattern was observed in human dermal fibroblasts (HDFs) following 48 h exposure (Figure 2b). Cell viability was 108.0%, 108.6%, 106.7%, 95.4%, and 81.8% following treatment with 2%, 5%, 10%, 20%, and 50% hs-VLNPs, respectively. These results indicate that hs-EVNP concentrations up to 20% did not substantially reduce cell viability in either of the human skin cell models under the respective exposure conditions.

Cytotoxicity was additionally evaluated in B16F10 murine melanoma cells and RAW264.7 murine macrophages using the exposure periods corresponding to their subsequent functional assays (Figure A2). In B16F10 cells exposed for 72 h, cell viability was 106.7%, 115.9%, 112.0%, and 104.4% at 2%, 5%, 10%, and 20% hs-VLNPs, respectively. In contrast, viability decreased to 49.7% at 50% hs-VLNPs (Figure A2a).

In RAW264.7 macrophages exposed for 24 h, cell viability was 101.8%, 104.3%, 111.8%, and 128.8% at hs-EVNP concentrations of 2%, 5%, 10%, and 20%, respectively. At 50%, however, cell viability markedly decreased to 12.6% (Figure A2b). Accordingly, hs-EVNP concentrations up to 20% were used to avoid overt cytotoxicity in subsequent experiments requiring this cell model.

Collectively, these results demonstrate cell type- and exposure time-dependent differences in the tolerance of hs-VLNPs. Concentrations up to 20% were generally well tolerated across HaCaT keratinocytes, HDFs, B16F10 cells, and RAW264.7 macrophages under their respective exposure conditions, whereas higher concentrations produced substantial reductions in viability in B16F10 and RAW264.7 cells. These cytotoxicity profiles were used to guide the selection of hs-EVNP concentrations for subsequent functional assays.

### 2.3. Antioxidant Activity of hs-VLNPs

The antioxidant activity of hs-VLNPs was evaluated using a DPPH radical scavenging assay. hs-VLNPs exhibited measurable radical scavenging activity across the tested concentration range, with the activity gradually increasing as the sample concentration increased (Figure A3a). Consistent with this trend, the ascorbic acid-equivalent antioxidant capacity also increased with increasing hs-EVNP concentration (Figure A3b). These results demonstrate concentration-dependent DPPH radical-scavenging activity of hs-VLNPs.

### 2.4. Effects of hs-VLNPs on Inflammatory Mediator Responses

The effects of hemp seed-derived vesicle-like nanoparticles (hs-VLNPs) on inflammatory mediator responses were evaluated using two complementary cellular models. Nitric oxide (NO) production was assessed in RAW264.7 murine macrophages, whereas TNF-α and IL-1β gene expression was evaluated in human dermal fibroblasts (HDFs) using quantitative real-time PCR (qRT-PCR).

In RAW264.7 macrophages, NO production increased from 0.42 μM in the untreated control to 14.24 μM following LPS stimulation (Figure 3a). Treatment with hs-VLNPs did not reduce LPS-associated NO production. Instead, NO levels increased to 16.5, 18.2, 22.5, and 29.8 μM following treatment with 5%, 10%, 20%, and 50% (*v*/*v*) hs-VLNPs, respectively. A progressive increase in NO production was therefore observed over the tested concentration range. However, because RAW264.7 cell viability was markedly reduced at 50% hs-VLNPs (Figure A2b), the NO response observed at this concentration should be interpreted with caution. The responses observed at concentrations up to 20%, at which cell viability was maintained, indicate that hs-VLNPs did not exert an inhibitory effect on NO production under the present experimental conditions.

Inflammatory cytokine gene expression was separately evaluated in HDFs stimulated with TNF-α/IFN-γ (10 ng/mL). TNF-α expression increased to 348.7% of the untreated control following stimulation. Treatment with 5%, 10%, and 20% hs-VLNPs resulted in TNF-α expression levels of 366.6%, 381.7%, and 416.8%, respectively (Figure 3b). Thus, hs-EVNP treatment did not attenuate TNF-α expression under these conditions and was associated with a concentration-related increase in TNF-α expression.

In contrast, IL-1β expression showed a different response pattern. TNF-α/IFN-γ stimulation increased IL-1β expression to 239.3% of the untreated control. Treatment with 5%, 10%, and 20% hs-VLNPs resulted in IL-1β expression levels of 233.8%, 211.1%, and 198.8%, respectively (Figure 3c). Thus, IL-1β expression progressively decreased with increasing hs-EVNP concentration, with the lowest level observed at 20%. Nevertheless, IL-1β expression remained above the unstimulated control level at all tested concentrations. Madecassoside, included as a reference treatment, did not reduce TNF-α expression under these conditions, whereas it lowered IL-1β expression relative to the stimulated control (Figure 3b,c).

Collectively, hs-VLNPs exhibited distinct mediator- and cell context-dependent effects on inflammatory responses. Within non-cytotoxic concentration ranges, NO production in RAW264.7 macrophages was not suppressed, while TNF-α expression in HDFs increased and IL-1β expression was partially attenuated with increasing hs-EVNP concentration. These findings do not support a generalized anti-inflammatory effect of hs-VLNPs; rather, they indicate differential modulation of individual inflammatory mediators depending on the cellular context and inflammatory endpoint examined.

### 2.5. hs-VLNPs Promote Procollagen Production and Attenuate UVB-Induced MMP-1 Expression

The effects of hs-VLNPs on collagen homeostasis-related responses were evaluated by examining procollagen type I production and UVB-induced matrix metalloproteinase-1 (MMP-1) expression in human dermal fibroblasts (HDFs).

As shown in Figure 4a, hs-EVNP treatment increased procollagen type I production in a concentration-dependent manner. Procollagen levels increased from 30.9 ng/mL in the untreated control to 34.2, 36.7, 42.6, and 44.8 ng/mL following treatment with 1%, 2%, 5%, and 10% (*v*/*v*) hs-VLNPs, respectively. The highest level was observed at 10% hs-VLNPs, corresponding to an approximately 45% increase relative to the untreated control (Figure 4a).

The effects of hs-VLNPs on a collagen degradation-related response were further evaluated by measuring MMP-1 gene expression following UVB irradiation. UVB exposure (20 mJ/cm^2^) increased MMP-1 expression to 251.6% of the non-irradiated control (Figure 4b). Treatment with hs-VLNPs progressively attenuated this UVB-induced increase, with MMP-1 expression decreasing to 226.8%, 172.5%, 133.6%, and 120.2% following treatment with 2%, 5%, 10%, and 20% hs-VLNPs, respectively. TGF-β (10 ng/mL), included as a reference treatment, resulted in an MMP-1 expression level of 153.9% of the non-irradiated control. The MMP-1 expression level observed at 20% hs-VLNPs (120.2%) was numerically lower than that observed in the TGF-β-treated reference group (153.9%) (Figure 4b).

Collectively, these findings show that hs-VLNPs increased procollagen type I production while attenuating UVB-induced MMP-1 expression in human dermal fibroblasts. The coordinated modulation of these two collagen-related processes suggests that hs-VLNPs may influence both matrix synthesis and degradation-associated responses under the present in vitro conditions.

### 2.6. hs-VLNPs Enhance Scratch Closure in Human Keratinocytes

The effect of hemp seed-derived vesicle-like nanoparticles (hs-VLNPs) on keratinocyte scratch closure was evaluated using an in vitro scratch assay. Following scratch formation, cells were treated with hs-VLNPs at concentrations of 2%, 5%, 10%, and 20% (*v*/*v*), and the remaining scratch area was measured after 24 h relative to the untreated control.

As shown in Figure 5, hs-EVNP treatment progressively reduced the remaining scratch area compared with the untreated control. Relative scratch area decreased from 100% in the control group to approximately 90%, 89%, 87%, and 83% following treatment with 2%, 5%, 10%, and 20% hs-VLNPs, respectively. The greatest reduction was observed at 20% hs-VLNPs, corresponding to an approximately 17% decrease in the remaining scratch area relative to the untreated control. Madecassoside (20 ppm), included as a reference treatment, resulted in a relative scratch area of approximately 89%.

Collectively, these results indicate that hs-VLNPs enhanced scratch closure in a concentration-dependent manner under the present experimental conditions. The reduction in remaining scratch area is consistent with enhanced keratinocyte migratory activity, although the relative contributions of cell migration and proliferation were not independently determined in this assay.

### 2.7. Effects of hs-VLNPs on Melanogenesis and Tyrosinase Activity

The effects of hemp seed-derived vesicle-like nanoparticles (hs-VLNPs) on melanogenesis were evaluated by measuring intracellular melanin production in B16F10 murine melanoma cells. Compared with the α-MSH-stimulated control, which was normalized to 100%, treatment with hs-VLNPs reduced melanin production across the tested concentration range (Figure 6a). Melanin production decreased to approximately 69% at 2% hs-VLNPs and was further reduced to approximately 63%, 59%, and 58% following treatment with 5%, 10%, and 20% hs-VLNPs, respectively. Because B16F10 cell viability was maintained at concentrations up to 20% under the corresponding 72 h exposure condition (Figure A2a), the observed reduction in melanin production was not attributable to overt cytotoxicity within this concentration range.

To determine whether the reduction in cellular melanin production was associated with direct inhibition of tyrosinase enzymatic activity, a cell-free mushroom tyrosinase assay was performed. In contrast to the cellular melanogenesis assay, hs-VLNPs exhibited little direct inhibitory activity against tyrosinase at concentrations of 2–20% (Figure 6b). Thus, the reduction in melanin production observed in B16F10 cells was not accompanied by a corresponding inhibition of tyrosinase activity in the cell-free assay.

Ethyl ascorbyl ether was included as a reference compound to verify the responsiveness of the tyrosinase assay. Ethyl ascorbyl ether produced a concentration-dependent increase in tyrosinase inhibition, reaching approximately 60% inhibition at 20,000 ppm (Figure 6c). This response confirmed that the assay conditions were capable of detecting direct inhibition of tyrosinase activity.

Collectively, these results show that hs-VLNPs reduced cellular melanin production without appreciably inhibiting tyrosinase activity in the cell-free assay. The difference between the cellular melanogenesis and direct enzyme assays suggests that the effect of hs-VLNPs on melanin production may involve cellular regulatory mechanisms rather than direct inhibition of tyrosinase catalytic activity. Further mechanistic studies will be required to identify the pathways involved.

## 3. Materials and Methods

### 3.1. Isolation of Hemp Seed-Derived Vesicle-like Nanoparticles (hs-VLNPs)

Isolation of extracellular vesicle-like nanoparticles (EVNPs) from plant-derived materials presents inherent technical challenges due to the presence of abundant particulate components, including cell wall fragments, storage proteins, fibrous materials, and nanoscale insoluble particles. These components can interfere with conventional isolation approaches such as ultracentrifugation and tangential flow filtration (TFF), frequently resulting in membrane fouling and reduced processing efficiency. In particular, fibrous impurities from plant tissues can rapidly obstruct TFF membranes and may not be readily removed by simple washing, thereby limiting membrane reusability and process robustness.

In addition, plant-derived samples are often processed in relatively large volumes, which can impose practical limitations on centrifugation-based pre-treatment. Processing large sample volumes generally requires high-capacity centrifugation systems, whereas the absence of such equipment necessitates repeated batch processing, thereby increasing processing time and operational complexity. In the present study, the workflow was designed for in vitro experimental purposes, and the sample volume was scaled to enable processing using standard laboratory centrifugation equipment. Accordingly, a multi-step workflow combining pre-clarification, sequential size-based filtration, electrokinetic capture, and TFF was employed to facilitate enrichment and downstream processing of vesicle-like nanoparticles, as summarized in Figure 1a.

As illustrated in Figure 1a, dehulled hemp seeds (*Cannabis sativa* L.) were washed with purified water and homogenized in phosphate-buffered saline (PBS) using a high-speed blender. The homogenate was first passed through a coarse mesh to remove large insoluble materials and residual seed coat fragments. The resulting suspension was centrifuged at 4000× *g* for 30 min at 4 °C, after which the intermediate aqueous fraction was carefully collected while excluding the upper lipid-rich layer and the bottom pellet. The recovered fraction was subsequently centrifuged at 7000× *g* for 60 min at 4 °C, and the intermediate aqueous fraction was again collected while excluding the upper lipid-rich layer and sedimented material. The recovered aqueous fraction was then sequentially passed through 0.8-μm and 0.45-μm membrane filters to further remove residual micron-sized particles and particulate materials prior to ExoFilter [20] processing (Figure 1a). Despite these pre-treatment steps, direct application of TFF remained susceptible to membrane fouling, presumably because nanoscale particulate components remained in the clarified plant extract. Therefore, an electrokinetic capture step was introduced before TFF processing.

During the electrokinetic capture step, negatively charged vesicle-like particles were preferentially retained on a positively charged mesh matrix through electrostatic interactions under controlled flow conditions [20]. The retained particles were subsequently eluted using an optimized buffer designed to disrupt these electrostatic interactions, thereby generating an enriched vesicle-like nanoparticle fraction (Figure 1a). The electrokinetic capture procedure is described in further detail in Section 2.2.

The recovered eluate was subsequently subjected to TFF using a 50 nm membrane (TFF-Easy, HansaBiomed, Tallin, Estonia) for further concentration and buffer exchange (Figure 1a). Additional TFF processing was performed as required to obtain the desired final sample volume. The resulting preparation, herein referred to as hemp seed-derived extracellular vesicle-like nanoparticles (hs-VLNPs), was collected and stored at 4 °C for short-term use or −80 °C for long-term storage until physicochemical characterization and subsequent in vitro analyses.

### 3.2. Electrokinetic Isolation of EV-like Nanoparticles

To improve the processing of pre-clarified hemp seed suspensions prior to TFF, an electrokinetic filtration step was incorporated using a proprietary electrokinetic capture system, ExoFilter (Microgentas, Seoul, Republic of Korea) developed in our laboratory [20]. The system contains a positively charged mesh matrix that facilitates charge-mediated retention of negatively charged vesicle-like particles from clarified suspensions.

As illustrated in Figure 1a, the filtered hemp seed extract was passed through the ExoFilter unit under controlled flow conditions. Negatively charged vesicle-like particles were preferentially retained on the cationic mesh through electrostatic interactions, whereas weakly interacting and non-retained components passed through the device. This step reduced the particulate load entering the subsequent membrane-filtration stage.

The retained particles were then eluted using an optimized buffer designed to disrupt electrostatic interactions between the vesicle-like particles and the mesh surface. The capture–elution process also enabled partial volume reduction, with the recovered fraction concentrated to approximately one-fifth of the initial processed volume. The resulting eluate was subsequently subjected to TFF for further concentration and buffer exchange.

### 3.3. Physicochemical Characterization of EV-like Nanoparticles

The morphology of the isolated hemp seed-derived vesicle-like nanoparticles (hs-VLNPs) was examined using cryogenic transmission electron microscopy (cryo-TEM). Samples were vitrified on lacey carbon grids and imaged to assess particle morphology and the presence of membrane-enclosed vesicular structures (Figure 1b).

Particle size distribution and concentration were analyzed using a nanoparticle tracking analysis system (ZetaView-QUATT, Particle Metrix, Ammersee, Germany). Samples were diluted in particle-free buffer to obtain particle counts within the recommended detection range. Hydrodynamic particle size and particle concentration were calculated from multiple recorded videos using the instrument software (Figure 1c).

For zeta potential analysis, hs-EVNP samples were diluted in a low-conductivity buffer and analyzed using the same ZetaView system in electrophoretic measurement mode. Zeta potential values were obtained from repeated measurements, and measurements were performed in triplicate (Appendix A Figure A1a). Total protein concentration of the isolated hs-VLNP preparation was determined using a bicinchoninic acid (BCA) assay according to the manufacturer’s instructions. The particle-to-protein ratio was calculated by dividing the particle concentration determined by NTA by the total protein concentration (Appendix A Figure A1b).

### 3.4. Cell Culture

Human keratinocytes (HaCaT), human dermal fibroblasts (HDFs), B16F10 murine melanoma cells, and RAW264.7 murine macrophages were used in this study. HaCaT cells were used for the assessment of cell viability and scratch closure. HDFs were used for the evaluation of cell viability, inflammatory cytokine gene expression, procollagen production, and UVB-induced MMP-1 expression. B16F10 cells were used for the assessment of cell viability and melanogenesis, whereas RAW264.7 cells were used for the assessment of cell viability and nitric oxide (NO) production.

Cells were maintained in their respective culture media under standard culture conditions at 37 °C in a humidified atmosphere containing 5% CO_2_. Prior to each assay, cells were seeded at appropriate densities and allowed to attach before treatment according to the assay-specific experimental conditions described below.

### 3.5. Cell Viability Assay

The cytotoxicity of hemp seed-derived vesicle-like nanoparticles (hs-VLNPs) was evaluated using a CCK-8-based cell viability assay in HaCaT keratinocytes, HDFs, B16F10 murine melanoma cells, and RAW264.7 murine macrophages. Cells were seeded in 96-well plates at appropriate densities and cultured for 24 h to allow cell attachment, followed by serum starvation for 1 h prior to treatment.

The hs-VLNP preparation, suspended in PBS, was diluted directly in serum-free culture medium to the indicated concentrations. For each treatment concentration, the corresponding control wells received an equal volume of PBS as a volume-matched vehicle control. The stock hs-EVNP preparation contained approximately 1.7 × 10^10^ particles/mL, as determined by nanoparticle tracking analysis (NTA). Based on the stock particle concentration, hs-EVNP concentrations of 2%, 5%, 10%, 20%, and 50% (*v*/*v*) corresponded to approximately 3.4 × 10^8^, 8.5 × 10^8^, 1.7 × 10^9^, 3.4 × 10^9^, and 8.5 × 10^9^ particles/mL, respectively. These concentrations were used for the cell viability assays in HaCaT cells, HDFs, B16F10 cells, and RAW264.7 cells.

The exposure period differed according to the cell type and corresponding experimental conditions. HaCaT and RAW264.7 cells were treated with hs-VLNPs for 24 h, HDFs for 48 h, and B16F10 cells for 72 h. Cell viability in HaCaT cells and HDFs is presented in Figure 2, whereas the corresponding viability data for B16F10 and RAW264.7 cells are provided in Appendix A Figure A2.

Following treatment, cell viability was determined using a CCK-8 assay according to the test protocol. Absorbance was measured using a microplate reader, and cell viability was expressed as a percentage relative to the PBS-treated control.

Cell viability was calculated using the following equation:

Cell viability (%) = (*A_s_* − *A_b_*)/(*A_c_* − *A_b_*) × 100

where *A_s_* is the absorbance of the sample, *A_b_* is the absorbance of the blank, and *A_c_* is the absorbance of the control. All experiments were performed in triplicate.

### 3.6. Scratch Closure Assay

The effect of hs-VLNPs on cell migration and scratch closure was evaluated using an in vitro scratch assay in HaCaT keratinocytes. Cells were seeded in culture plates and grown to near confluence. A linear scratch was created across the cell monolayer using a sterile pipette tip, and detached cells were removed by washing with phosphate-buffered saline (PBS).

HaCaT cells were subsequently treated with hs-VLNPs at the indicated concentrations and incubated for 24 h. Images of the scratched area were captured immediately after scratching (0 h) and after 24 h of treatment using an inverted microscope equipped with a digital camera. The remaining scratch area was quantified using ImageJ software (version 1.54p, National Institutes of Health, Bethesda, MD, USA).

Scratch closure was calculated relative to the initial scratch area as follows:

Scratch closure (%) = (A_0_ − A_24_)/(A_0_) × 100

where A_0_ represents the scratch area at 0 h and A_24_ represents the remaining scratch area after 24 h of treatment.

### 3.7. Inflammatory Mediator Analysis

#### 3.7.1. Nitric Oxide Production in RAW264.7 Macrophages

Nitric oxide (NO) production was evaluated in RAW264.7 murine macrophages. Cells were seeded at an appropriate density and cultured for 24 h, followed by serum starvation for 1 h. The cells were stimulated with lipopolysaccharide (LPS) and treated with hs-VLNPs at the indicated concentrations for 24 h. Untreated cells and LPS-stimulated cells without hs-EVNP treatment were used as the unstimulated and stimulated controls, respectively.

Following treatment, the culture supernatants were collected, and NO production was evaluated by quantifying nitrite accumulation using a colorimetric NO assay. Absorbance was measured according to the assay protocol, and nitrite concentrations were calculated from the corresponding standard curve and expressed as μM.

#### 3.7.2. Inflammatory Cytokine Gene Expression in Human Dermal Fibroblasts

The effects of hs-VLNPs on inflammatory cytokine gene expression were evaluated in human dermal fibroblasts (HDFs) using quantitative real-time PCR (qRT-PCR). Cells were seeded at an appropriate density and cultured for 12 h, followed by serum starvation for 1 h. The cells were stimulated with TNF-α/IFN-γ (10 ng/mL) and treated with hs-VLNPs at concentrations of 5%, 10%, and 20% (*v*/*v*) for 24 h. Madecassoside (100 μg/mL) was included as a reference treatment.

Untreated cells and cytokine-stimulated cells without test material (0% hs-EVNP) served as the unstimulated and stimulated (induced) controls, respectively. Relative TNF-α and IL-1β expression in all groups was expressed as fold change relative to the untreated control, whereas the effect of each treatment was assessed by comparison with the stimulated (induced) control.

Following treatment, the culture medium was removed, and the cells were washed with phosphate-buffered saline (PBS). Total RNA was extracted and reverse-transcribed into complementary DNA (cDNA). Quantitative real-time PCR was performed using a QuantStudio 1 Real-Time PCR System (Applied Biosystems, Foster City, CA, USA). The PCR conditions consisted of UDG activation at 50 °C for 2 min and Dual-Lock™ DNA polymerase activation at 95 °C for 2 min, followed by 40 cycles of denaturation at 95 °C for 15 s, annealing at 55–60 °C for 15 s, and extension at 72 °C for 1 min.

The expression levels of TNF-α and IL-1β were normalized to GAPDH as the housekeeping gene, and relative gene expression was calculated using the 2^−ΔΔCt^ method. Primer sequences used for qRT-PCR are provided in Appendix A Table A1.

### 3.8. Collagen Production Assay

The effect of hs-VLNPs on procollagen production was evaluated in human dermal fibroblasts (HDFs) using an enzyme-linked immunosorbent assay (ELISA). Cells were seeded at an appropriate density and cultured for 12 h, followed by serum starvation for 1 h. The cells were then treated with hs-VLNPs at concentrations of 1%, 2%, 5%, and 10% (*v*/*v*) for 48 h under standard culture conditions. PBS-treated cells were used as the control.

Following treatment, the culture supernatants were collected and centrifuged to remove cells and residual particulate material. Procollagen type I C-peptide (PIP) levels in the resulting supernatants were quantified using a commercial PIP ELISA kit according to the manufacturer’s instructions.

Absorbance was measured using a microplate reader, and PIP concentrations were calculated from a standard calibration curve and expressed as ng/mL. Measurements were performed in triplicate.

### 3.9. MMP-1 Gene Expression Analysis

The effect of hemp seed-derived vesicle-like nanoparticles (hs-VLNPs) on UVB-induced matrix metalloproteinase-1 (MMP-1) gene expression was evaluated in human dermal fibroblasts (HDFs) using quantitative real-time PCR (qRT-PCR). Cells were seeded at an appropriate density and cultured for 12 h, followed by serum starvation for 1 h.

Cells assigned to the UVB-treated groups were irradiated with UVB at 20 mJ/cm^2^ and subsequently treated with hs-VLNPs at concentrations of 2%, 5%, 10%, and 20% (*v*/*v*) for 24 h. Non-irradiated cells served as the baseline control, whereas UVB-irradiated cells without hs-EVNP treatment served as the UVB-stimulated control. TGF-β (10 ng/mL) was included as a reference treatment.

Following treatment, the cells were processed for total RNA extraction, reverse transcription, and qRT-PCR as described in Section 3.7. The PCR conditions consisted of UDG activation at 50 °C for 2 min and Dual-Lock™ DNA polymerase activation at 95 °C for 2 min, followed by 40 cycles of denaturation at 95 °C for 15 s, annealing at 55–60 °C for 15 s, and extension at 72 °C for 1 min.

MMP-1 expression was normalized to GAPDH as the housekeeping gene, and relative gene expression was calculated using the 2^−ΔΔCt^ method. Primer sequences are provided in Appendix A Table A1.

### 3.10. Antioxidant Activity Assay

The antioxidant activity of hs-VLNPs was evaluated using a DPPH (1,1-diphenyl-2-picrylhydrazyl) free radical scavenging assay. hs-EVNP samples were tested at concentrations of 10%, 20%, 50%, 75%, and 100% (*w*/*v*), as specified in the original assay protocol.

A DPPH solution was prepared by dissolving 0.4 mg of DPPH in 50 mL of methanol and stored at −20 °C in the dark until use. For the sample blank, 50 μL of hs-EVNP sample was mixed with 150 μL of methanol. For the DPPH reaction, 50 μL of hs-EVNP sample was mixed with 150 μL of DPPH solution and incubated at room temperature in the dark for 30 min.

Absorbance was measured at 520 nm using a UV–Vis spectrophotometer (AMR-100, ALLSHENG, Hangzhou, China). DPPH radical scavenging activity (RSA) was calculated as follows:

$$\mathrm{DPPH}\;\mathrm{RSA}\;(\%)\;=\;\left[1-\left({ABS}_{s}-\;{ABS}_{s,blank}\right)/\left({ABS}_{c}-\;{ABS}_{c,blank}\right)\right]\times 100$$

where *ABS_s_* is the absorbance of the sample mixed with DPPH solution, *ABS*_*s*,*blank*_ is the absorbance of the corresponding sample blank without DPPH, *ABS_c_* is the absorbance of the control containing DPPH without sample, and *ABS*_*c*,*blank*_ is the absorbance of the corresponding control blank without DPPH.

In addition, ascorbic acid was used to generate a standard calibration curve, and antioxidant capacity was expressed as ascorbic acid equivalents (AE, μM) per 200 μL sample.

### 3.11. Melanin Production Assay

B16F10 cells were stimulated with α-MSH (100 nM) and treated with hs-VLNPs at the indicated concentrations for 72 h. After incubation, intracellular melanin content was quantified as described below.

The effect of hs-VLNPs on melanogenesis was evaluated by measuring intracellular melanin content in B16F10 murine melanoma cells. Cells were seeded at an appropriate density and cultured for 24 h, followed by serum starvation for 1 h. The cells were then treated with hs-VLNPs at the indicated concentrations for 72 h under standard culture conditions.

Following treatment, the cells were harvested and incubated with 1 N NaOH at 60 °C for 1 h to solubilize intracellular melanin. Absorbance was subsequently measured using a microplate reader, and melanin production was expressed relative to the control group.

Melanin production was calculated as follows:

$$\mathrm{Melanin}\;\mathrm{production}\;(\%)\;=\;\left(A_{s}-A_{b})/(A_{c}-A_{b}\right)\;\times \;100$$

where *A_s_* is the absorbance of the treated sample, *A_b_* is the absorbance of the blank, and *A_c_* is the absorbance of the control. All experiments were performed in triplicate.

### 3.12. Tyrosinase Inhibition Assay

The direct inhibitory effect of hs-VLNPs on tyrosinase activity was evaluated using a mushroom tyrosinase assay. hs-EVNP samples were prepared at the indicated concentrations in 0.1 M sodium phosphate buffer (pH 6.5). Mushroom tyrosinase and L-tyrosine were used as the enzyme and substrate, respectively.

The reaction mixture containing sodium phosphate buffer, hs-EVNP sample, and mushroom tyrosinase was prepared in a 96-well plate. The enzymatic reaction was initiated by the addition of L-tyrosine and incubated at 37 °C. Following incubation, tyrosinase activity was determined by measuring absorbance at 490 nm using a microplate reader.

Tyrosinase inhibition was calculated as follows:

$$\mathrm{Tyrosinase}\;\mathrm{inhibition}\;(\%)\;=\;100\;-\;[\left(B-B^{\prime })/(A-A^{\prime })\;\times \;100\right]$$

where *A* represents the absorbance of the control reaction containing enzyme and substrate, *B* represents the absorbance of the reaction containing enzyme, substrate, and sample, and *A*′ and *B*′ represent the corresponding absorbance values measured without enzyme.

### 3.13. Statistical Analysis

Quantitative data are presented as mean ± standard deviation (SD) of triplicate technical wells. Technical replicates were used to characterize within-assay variability and were not treated as independent biological replicates for inferential statistical testing. Accordingly, no *p*-values or statistical significance claims were derived from the technical replicates, and the results were interpreted descriptively based on the observed effect magnitudes and concentration-dependent trends.

## 4. Discussion

Plant-derived extracellular vesicles and vesicle-like nanoparticles have attracted increasing interest as naturally occurring nanomaterials capable of carrying diverse biological cargo and modulating cellular responses. Nevertheless, their study remains complicated by substantial heterogeneity in plant sources, isolation procedures, particle identity, and quality-control criteria. In particular, vesicular particles recovered after disruption or homogenization of plant tissues cannot necessarily be regarded as bona fide extracellular vesicles because membrane fragments and other nanoscale structures generated during processing may coexist with naturally secreted vesicles [17]. In the present study, a nanoscale vesicular fraction was isolated from hemp seeds using a workflow combining sequential clarification, electrokinetic capture, and tangential flow filtration (TFF). Cryo-TEM revealed membrane-enclosed vesicular structures, while nanoparticle tracking analysis and zeta-potential measurements confirmed a nanoscale particle population with a negative surface charge. Because these characteristics support vesicle-like morphology but do not independently establish extracellular biogenesis, the isolated particles were conservatively designated hemp seed-derived vesicle-like nanoparticles (hs-VLNPs). Although the upstream centrifugation and filtration steps were designed to reduce lipid-rich seed-derived components, trace nanoscale lipid-rich particles cannot be completely excluded in the absence of composition-specific analyses. The electrokinetic capture step was incorporated to facilitate processing of complex plant homogenates prior to TFF, which is particularly relevant because fibrous materials, cell-wall fragments, and other particulate components can promote membrane fouling. Although the present workflow was performed at laboratory scale, its modular combination of clarification, charge-based enrichment, and membrane filtration may provide a practical basis for further process optimization and scale-up [21,22].

The biological findings indicate that hs-VLNPs exert a multidimensional rather than single-target cellular response. Cytotoxicity testing showed that concentrations up to 20% were generally well tolerated across the cell models used for subsequent functional assays under their respective exposure conditions, allowing the biological responses observed within this range to be interpreted without evidence of overt cytotoxicity. The highest hs-VLNP concentration used in most functional assays was 20% (*v*/*v*), corresponding nominally to approximately 3.4 × 10^9^ particles/mL and 39.4 μg/mL total protein based on the characterized stock preparation. Although direct comparison among plant-derived vesicular preparations is limited by differences in isolation methods and dose normalization, similar concentration ranges have been used in recent studies of *Centella asiatica*- and *Aloe vera*-derived nanovesicles in macrophage and skin fibroblast models [23,24].

The inflammatory data, however, did not support a generalized anti-inflammatory effect. In RAW264.7 macrophages, hs-VLNPs did not suppress LPS-induced NO production at concentrations up to 20%, which were not associated with overt cytotoxicity under the corresponding exposure conditions. In human dermal fibroblasts, TNF-α expression increased while IL-1β expression was partially attenuated. These divergent responses suggest mediator- and cell context-dependent modulation rather than uniform inflammatory suppression. Such complexity is consistent with the concept that plant-derived vesicular nanoparticles represent composite biological systems whose activity may reflect combined effects of membrane lipids, proteins, nucleic acids, and plant-specific metabolites [25]. Further investigation of iNOS, NF-κB, MAPK, and related signaling pathways will be required to clarify the mechanisms underlying these differential inflammatory responses. Anti-inflammatory activity has been reported for vesicular nanoparticles derived from several plant sources. For example, Allium tuberosum-derived nanovesicles reduced LPS-induced IL-1β, IL-6, TNF-α, and NF-κB-associated responses in RAW 264.7 macrophages, whereas *Centella asiatica*-derived exosome-like nanovesicles reduced nitric oxide production but showed more limited effects on other inflammatory markers. In comparison, hs-VLNPs did not uniformly suppress all inflammatory mediators in the present study, suggesting that the immunomodulatory effects of plant-derived vesicular nanoparticles may vary according to plant source, particle composition, cell type, and inflammatory stimulus [23,26].

A more coherent functional pattern emerged from the extracellular matrix-related, antioxidant, and scratch-closure assays. hs-VLNPs increased procollagen type I production while attenuating UVB-induced MMP-1 expression in human dermal fibroblasts, suggesting coordinated effects on processes related to collagen synthesis and degradation. Similar effects have been reported for other plant-derived vesicular nanoparticles; *Physalis peruviana*-derived exosome-like nanoparticles enhanced fibroblast migration and collagen I production while reducing MMP-1 [27], and kale-derived exosome-like nanovesicles increased type I collagen production through regulation of Smad7 [28].

In addition, hs-VLNPs exhibited concentration-dependent DPPH radical-scavenging activity in a cell-free assay. However, this finding does not establish intracellular antioxidant activity or a cellular protective mechanism. Intracellular ROS levels and redox- or stress-responsive signaling pathways, including Nrf2, MAPK/AP-1, and NF-κB, were not examined in the present study; therefore, the cellular mechanism underlying the observed UVB-related responses remains unknown. Future studies assessing intracellular ROS and relevant signaling pathways will be required to determine whether redox regulation contributes to the observed cellular effects of hs-VLNPs. Previous work on EV-like nanoparticles derived from *Cannabis sativa* adventitious roots reported modulation of MAPK and Nrf2 signaling [29]; however, these pathways were not evaluated in the present study.

Furthermore, hs-VLNPs enhanced scratch closure in HaCaT keratinocytes. Although this assay does not independently distinguish migration from proliferation and should not be interpreted as direct evidence of wound healing, the finding complements the collagen-related responses and suggests an influence on cellular processes associated with matrix maintenance and tissue remodeling. Similar migration- and wound-associated effects have been reported for vesicular nanoparticles derived from *Morinda officinalis* and *Houttuynia cordata* [12,30].

The melanogenesis experiments revealed a distinct functional profile. hs-VLNPs reduced α-MSH-induced melanin production in B16F10 cells at concentrations that maintained cell viability, whereas they produced little direct inhibition of mushroom tyrosinase activity in the cell-free assay. In contrast, ethyl ascorbyl ether showed the expected concentration-dependent tyrosinase inhibition, confirming the responsiveness of the enzymatic assay. The dissociation between reduced cellular melanin production and minimal inhibition of mushroom tyrosinase indicates that direct inhibition of tyrosinase catalytic activity is unlikely to fully explain the observed reduction in melanogenesis. However, the cellular mechanism underlying this effect remains undetermined. Previous studies have reported that plant-derived EVs from *Dendropanax morbifera* reduce melanogenesis together with MITF, tyrosinase, TRP-1, and TRP-2 expression [31], while EV-like nanoparticles derived from *Cannabis sativa* stems have also shown anti-melanogenic activity [32]. Future studies examining cellular tyrosinase activity and melanogenesis-related regulators, including MITF, TYRP1, and TYRP2, will be required to determine the cellular mechanism underlying the observed reduction in melanin production.

Taken together, the present findings are better interpreted as evidence of multifunctional modulation of skin-associated cellular processes than as a collection of independent cosmetic effects. The increase in procollagen type I production, attenuation of UVB-induced MMP-1 expression, enhancement of scratch closure, measurable antioxidant activity, and reduction in cellular melanin production collectively indicate that hs-VLNPs influence multiple processes associated with extracellular matrix maintenance, stress-related responses, and pigmentation regulation. At the same time, the heterogeneous inflammatory responses emphasize that the biological activity of hs-VLNPs is context dependent and should not be reduced to a broad anti-inflammatory claim. The emerging literature on vesicular nanoparticles from different tissues of *Cannabis sativa* further suggests that tissue origin may contribute to distinct but partially overlapping functional profiles. In this context, the present study extends previous work on stem- and adventitious root-derived nanoparticles by identifying hemp seeds as an additional source of biologically active *Cannabis sativa*-derived vesicle-like nanoparticles.

Several limitations remain. Although cryo-TEM, NTA, and zeta-potential measurements support the presence of vesicle-like nanoparticles, these analyses do not establish their extracellular biogenesis, and additional proteomic, lipidomic, RNA, and candidate marker analyses will be necessary to define their molecular identity more precisely [17]. The bioactive cargo responsible for the observed effects also remains unknown, and comparison with non-vesicular hemp seed fractions will be important for determining whether these activities are specifically associated with the vesicular fraction. In addition, most biological experiments were performed in two-dimensional cell cultures, while the antioxidant and tyrosinase assays were cell-free; therefore, three-dimensional skin equivalents, ex vivo models, and appropriately designed in vivo studies will be required to determine whether these effects translate to the tissue level. Batch-to-batch variability related to seed cultivar, cultivation conditions, harvest, and storage should also be addressed, together with reproducibility and process scale-up [22]. Nevertheless, the present study provides a basis for further investigation of hs-VLNPs as plant-derived nanomaterials with potential skin-related applications.

## 5. Conclusions

In this study, hemp seed-derived vesicle-like nanoparticles (hs-VLNPs) were isolated using an integrated workflow combining pre-clarification, sequential filtration, electrokinetic capture, and tangential flow filtration. The resulting nanoparticles exhibited membrane-enclosed vesicle-like morphology, a nanoscale size distribution, and a negative surface charge, supporting the enrichment of a vesicle-like nanoparticle fraction from a complex plant-derived matrix.

hs-VLNPs exhibited multifunctional biological activities associated with skin-related cellular processes, including increased procollagen type I production, attenuation of UVB-induced MMP-1 expression, enhanced scratch closure, measurable antioxidant activity, and reduced cellular melanin production. The reduction in melanin production occurred without appreciable direct inhibition of tyrosinase activity, suggesting that cellular regulatory mechanisms may be involved. Inflammatory responses were mediator- and cell context-dependent rather than uniformly suppressed, further indicating that hs-VLNPs exert complex biological effects rather than a generalized anti-inflammatory response.

Collectively, these findings provide a basis for further investigation of hemp seed-derived EVNPs as plant-derived nanomaterials for skin-related applications. Future studies addressing their molecular composition, biogenesis, mechanisms of action, batch-to-batch reproducibility, and efficacy in more physiologically relevant skin models will be important for defining their biological significance and translational potential.

## Figures and Tables

**Figure 1:**
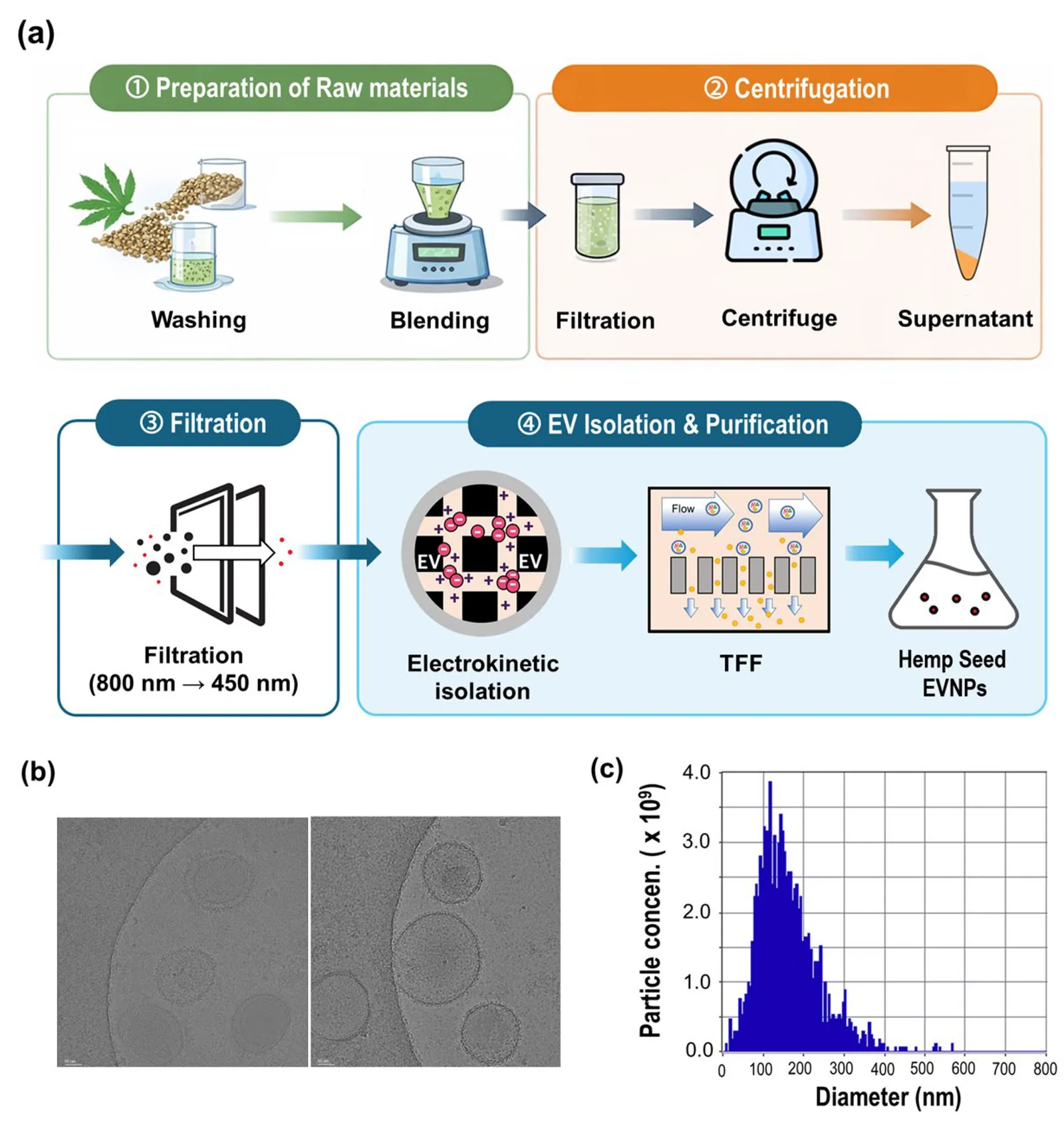
Isolation and physicochemical characterization of hemp seed-derived vesicle-like nanoparticles (hs-VLNPs). (**a**) Schematic overview of the isolation workflow, including sequential centrifugation at 4000× *g* and 7000× *g* with recovery of the intermediate aqueous fraction, sequential 0.8- and 0.45-μm filtration, electrokinetic charge-based capture using a cationic mesh (ExoFilter, Microgentas, Seoul, Republic of Korea), and subsequent tangential flow filtration (TFF) for buffer exchange and concentration; (**b**) cryo-TEM image showing intact vesicular morphology with well-defined membrane structures; (**c**) nanoparticle tracking analysis (NTA) illustrating the size distribution of the hs-VLNP population.

**Figure 2:**
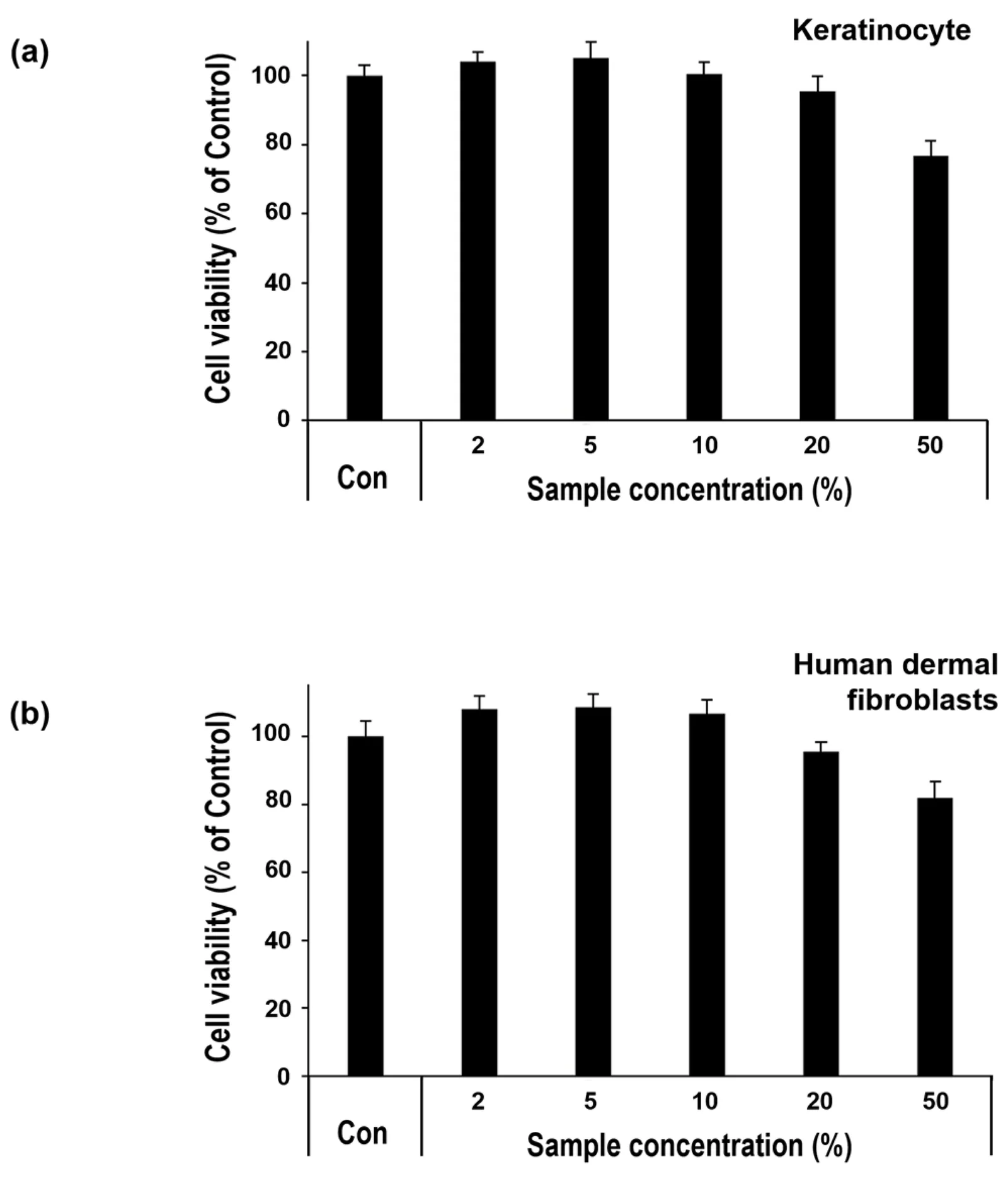
Cytotoxicity evaluation of hemp seed-derived vesicle-like nanoparticles (hs-VLNPs) in human skin cells. Cell viability was assessed using a CCK-8 assay after treatment with the indicated concentrations of hs-VLNPs for (**a**) 24 h in human keratinocytes (HaCaT) and (**b**) 48 h in human dermal fibroblasts (HDFs). Cell viability is expressed relative to the PBS-treated control (Con, 100%). Data are presented as mean ± standard deviation (SD) of triplicate technical wells. Error bars represent within-assay technical variability. The observed changes are presented descriptively, and no inferential statistical analysis was performed using technical replicates.

**Figure 3:**
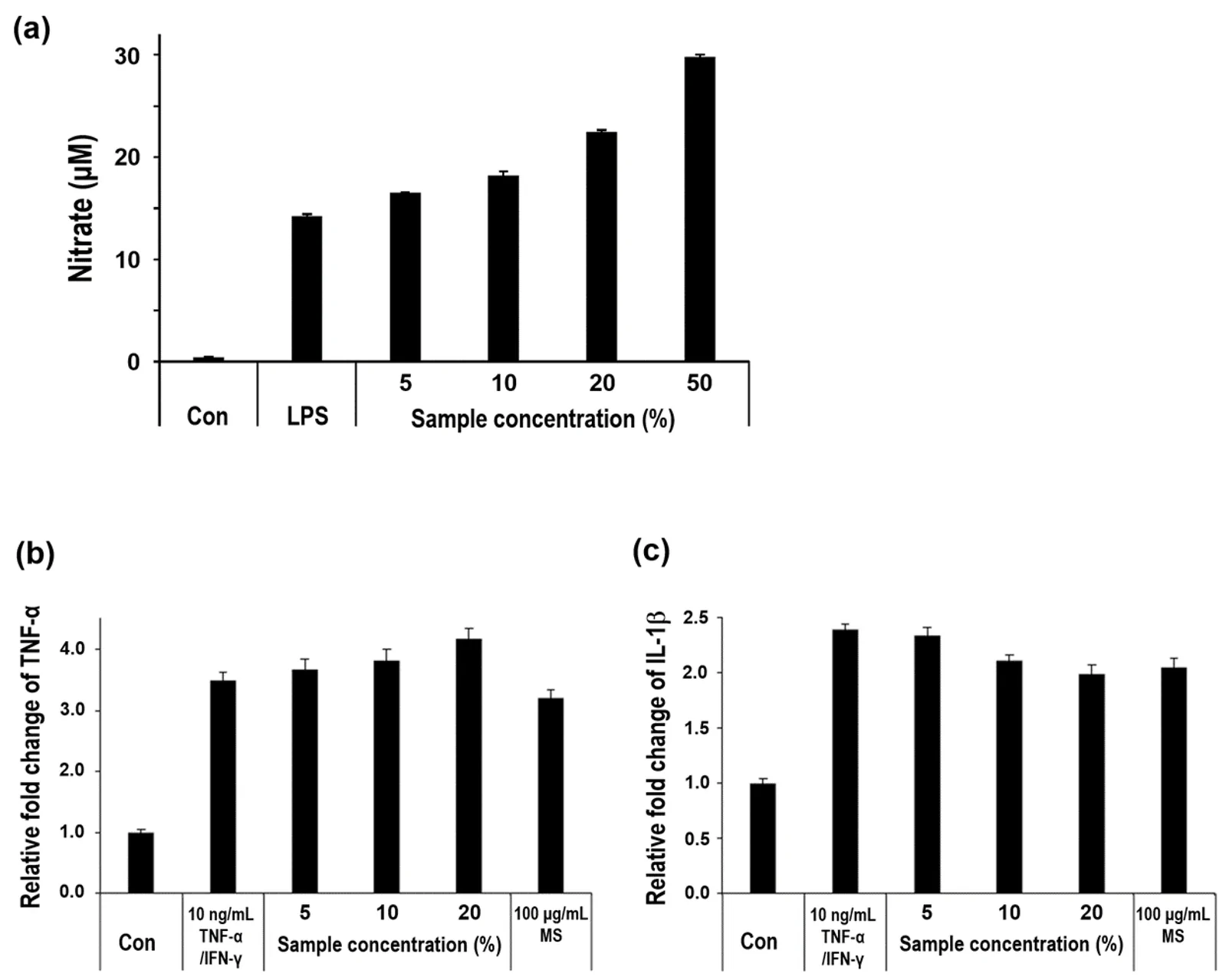
Effects of hemp seed-derived vesicle-like nanoparticles (hs-VLNPs) on inflammatory mediator responses. (**a**) Nitric oxide (NO) production in RAW264.7 murine macrophages following LPS stimulation and treatment with hs-VLNPs at the indicated concentrations. NO production was evaluated by quantifying nitrite accumulation using a colorimetric assay. (**b**) Relative TNF-α and (**c**) IL-1β mRNA expression in human dermal fibroblasts (HDFs) following stimulation with TNF-α/IFN-γ (10 ng/mL each) and treatment with hs-VLNPs at the indicated concentrations. TNF-α and IL-1β mRNA levels were determined by qRT-PCR and normalized to GAPDH. Con, untreated control; LPS, lipopolysaccharide-stimulated control; MS, madecassoside (100 μg/mL), included as a reference treatment. Data are presented as mean ± standard deviation (SD) of triplicate technical wells. Error bars represent within-assay technical variability. The observed changes are presented descriptively, and no inferential statistical analysis was performed using technical replicates.

**Figure 4:**
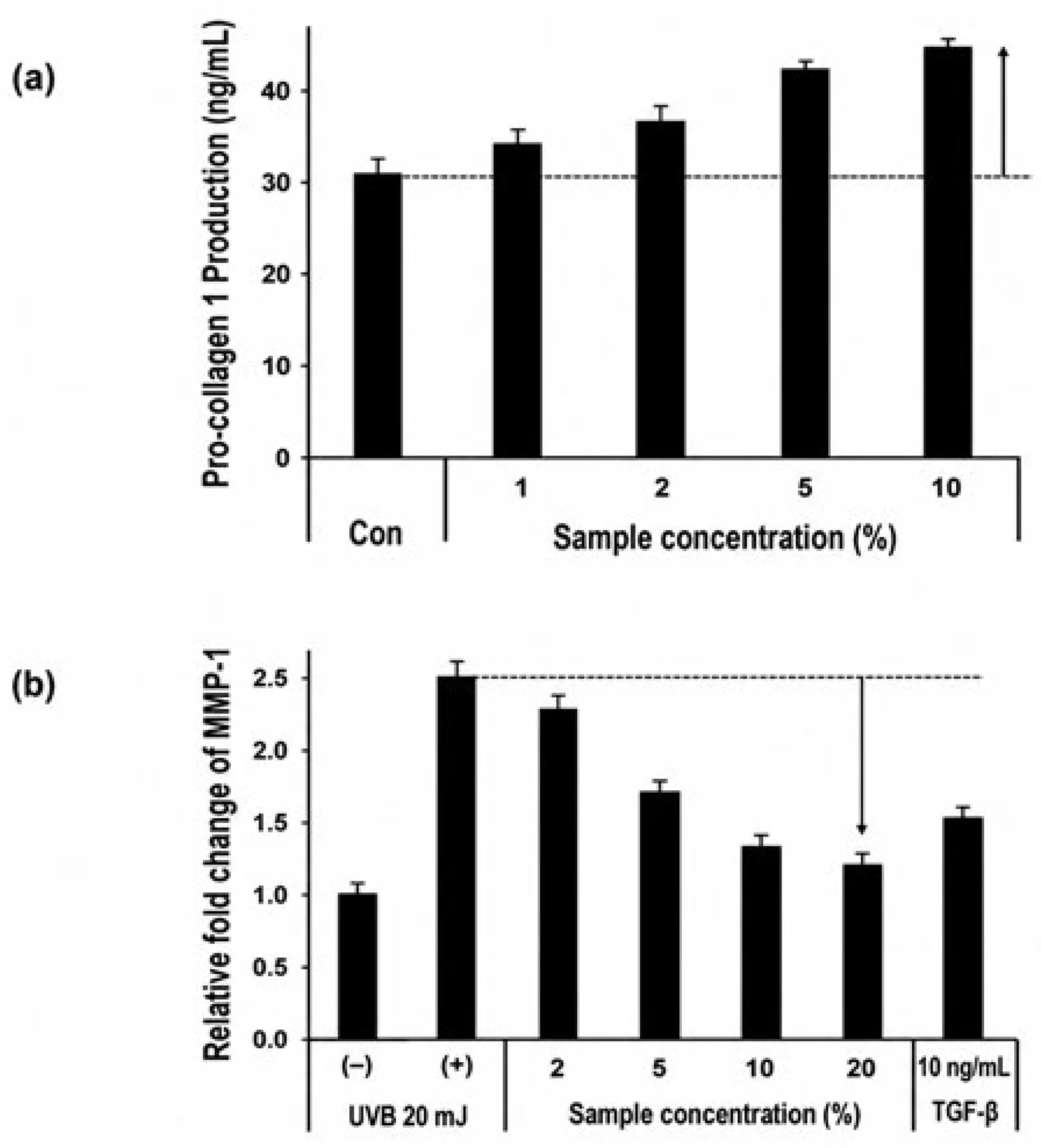
Effects of hemp seed-derived vesicle-like nanoparticles (hs-VLNPs) on procollagen production and UVB-induced MMP-1 expression in human dermal fibroblasts. (**a**) Procollagen type I production in HDFs following treatment with hs-VLNPs at the indicated concentrations, quantified by ELISA and expressed as ng/mL. (**b**) Relative MMP-1 mRNA expression in HDFs following UVB irradiation (20 mJ/cm^2^) and treatment with hs-VLNPs at the indicated concentrations, normalized to GAPDH and expressed relative to the non-irradiated control. (−), non-irradiated control; (+), UVB-irradiated control; TGF-β (10 ng/mL), included as a reference treatment. Data are presented as mean ± standard deviation (SD) of triplicate technical wells. Error bars represent within-assay technical variability. The observed changes are presented descriptively, and no inferential statistical analysis was performed using technical replicates.

**Figure 5:**
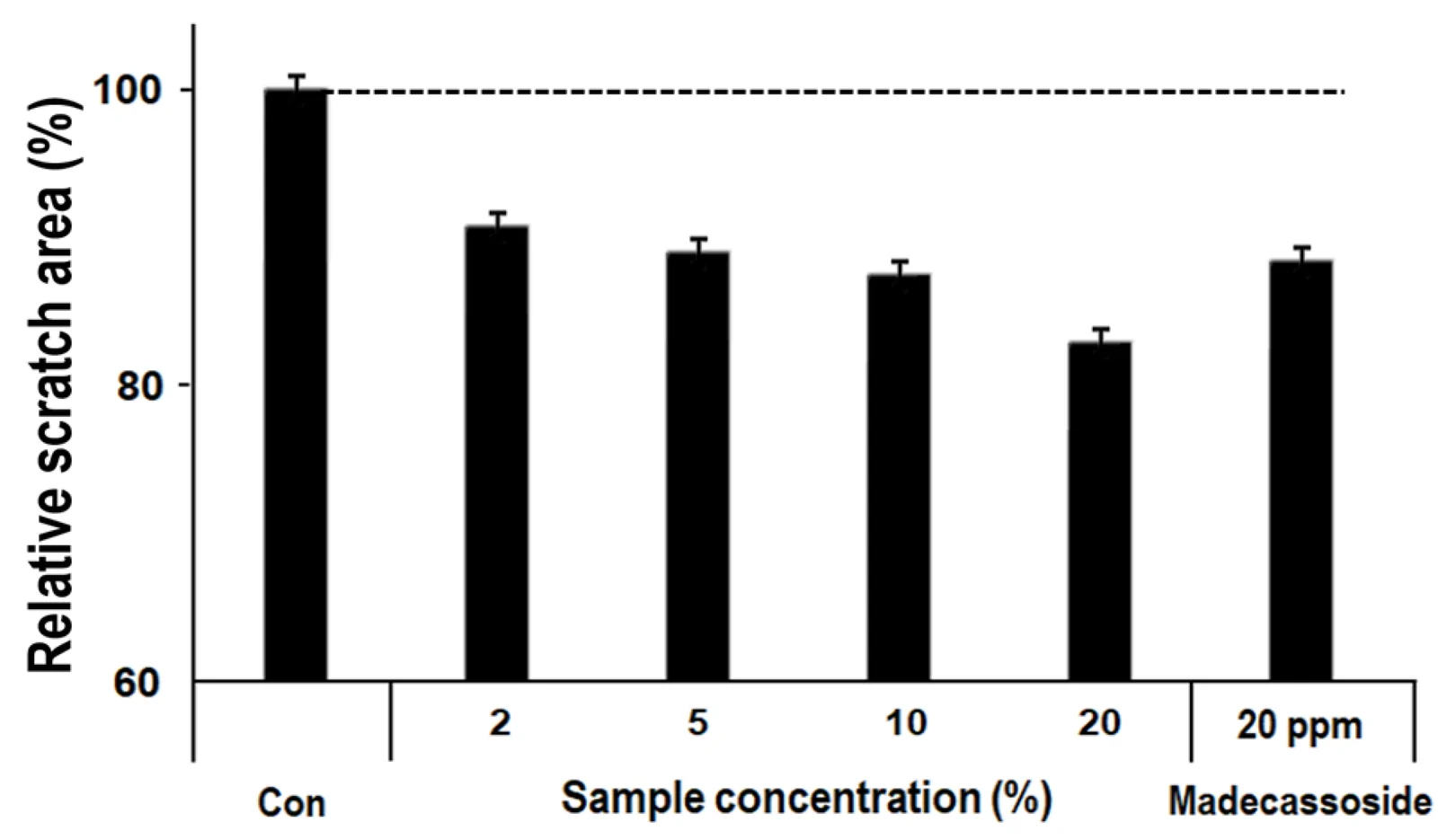
Effect of hemp seed-derived vesicle-like nanoparticles (hs-VLNPs) on scratch closure in human keratinocytes. HaCaT cells were subjected to an in vitro scratch assay and treated with hs-VLNPs at the indicated concentrations for 24 h. The remaining scratch area was measured and expressed relative to the untreated control (Con, 100%). Madecassoside (20 ppm) was included as a reference treatment. Data are presented as mean ± standard deviation (SD) of triplicate technical wells. Error bars represent within-assay technical variability. The observed changes are presented descriptively, and no inferential statistical analysis was performed using technical replicates.

**Figure 6:**
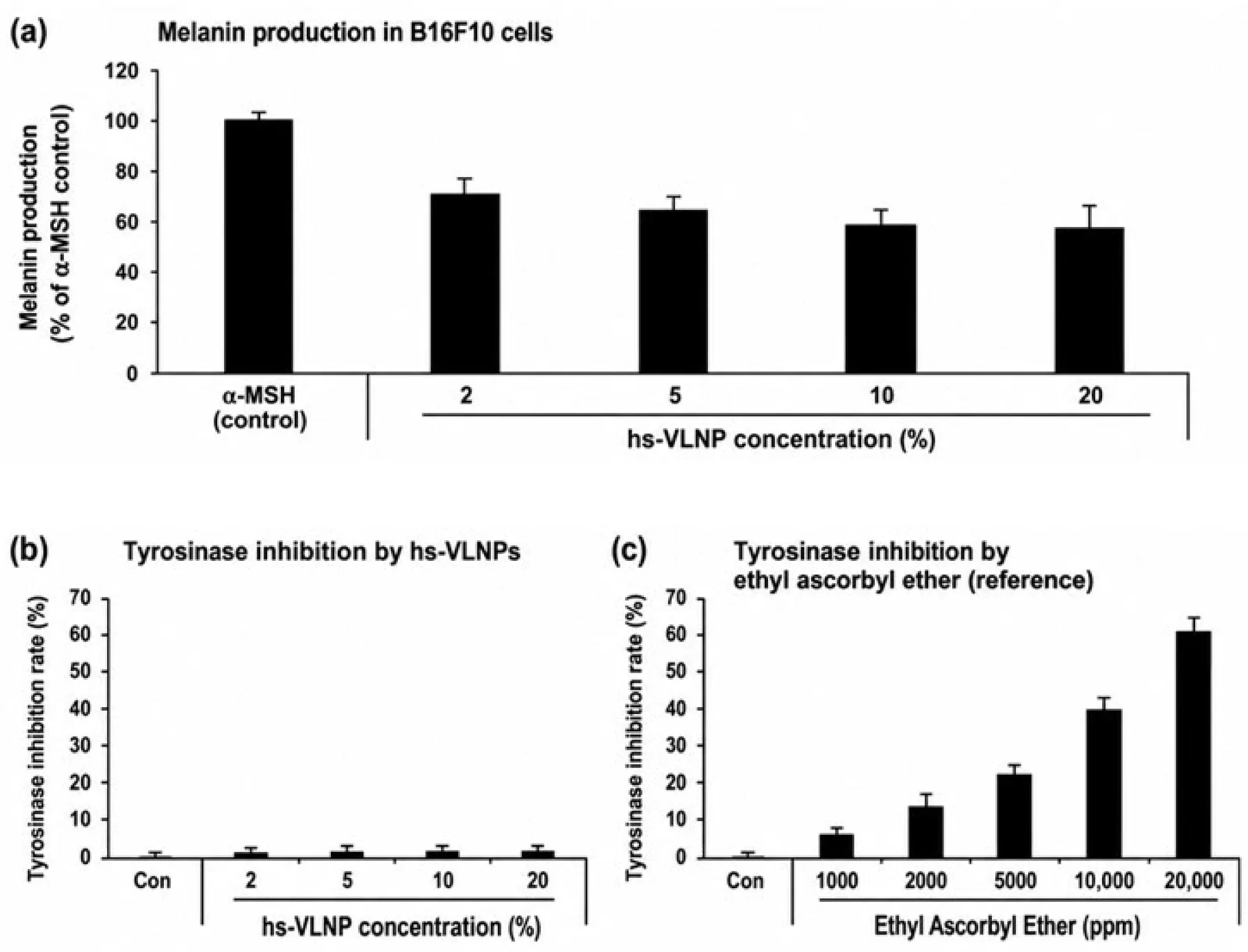
Effects of hemp seed-derived vesicle-like nanoparticles (hs-VLNPs) on melanin production and tyrosinase activity. (**a**) Intracellular melanin production in B16F10 murine melanoma cells stimulated with α-MSH (100 nM) and treated with hs-VLNPs at concentrations of 2–20% for 72 h. Melanin content was expressed relative to the α-MSH-stimulated control, which was normalized to 100%. (**b**) Direct tyrosinase inhibitory activity of hs-VLNPs at the indicated concentrations in a cell-free mushroom tyrosinase assay. (**c**) Tyrosinase inhibitory activity of ethyl ascorbyl ether, included as a reference compound, showing concentration-dependent inhibition and confirming the responsiveness of the assay. Con, control without treatment. Data are presented as mean ± standard deviation (SD) of triplicate technical wells. Error bars represent within-assay technical variability. The observed changes are presented descriptively, and no inferential statistical analysis was performed using technical replicates.

**Figure A1:**
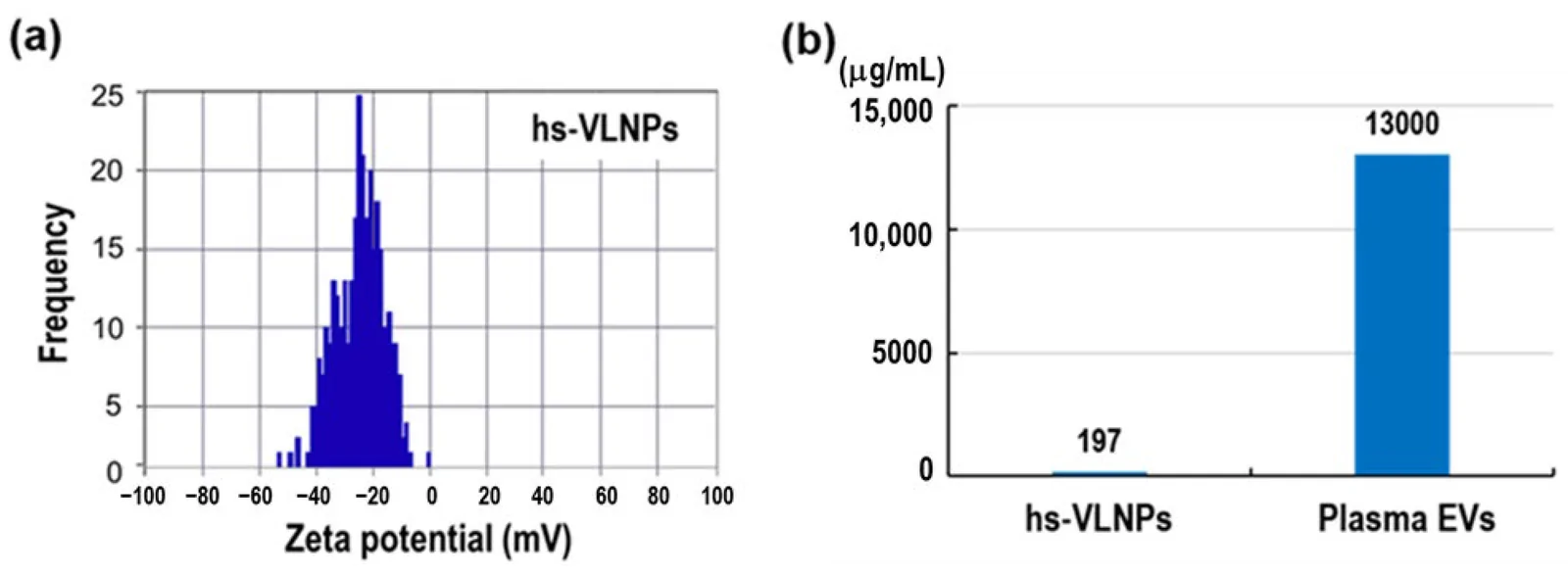
Additional physicochemical characterization of hs-VLNPs. (**a**) Representative NTA particle-size distribution of the isolated hs-VLNP preparation. (**b**) Total protein concentration determined by BCA assay (197 μg/mL).

**Figure A2:**
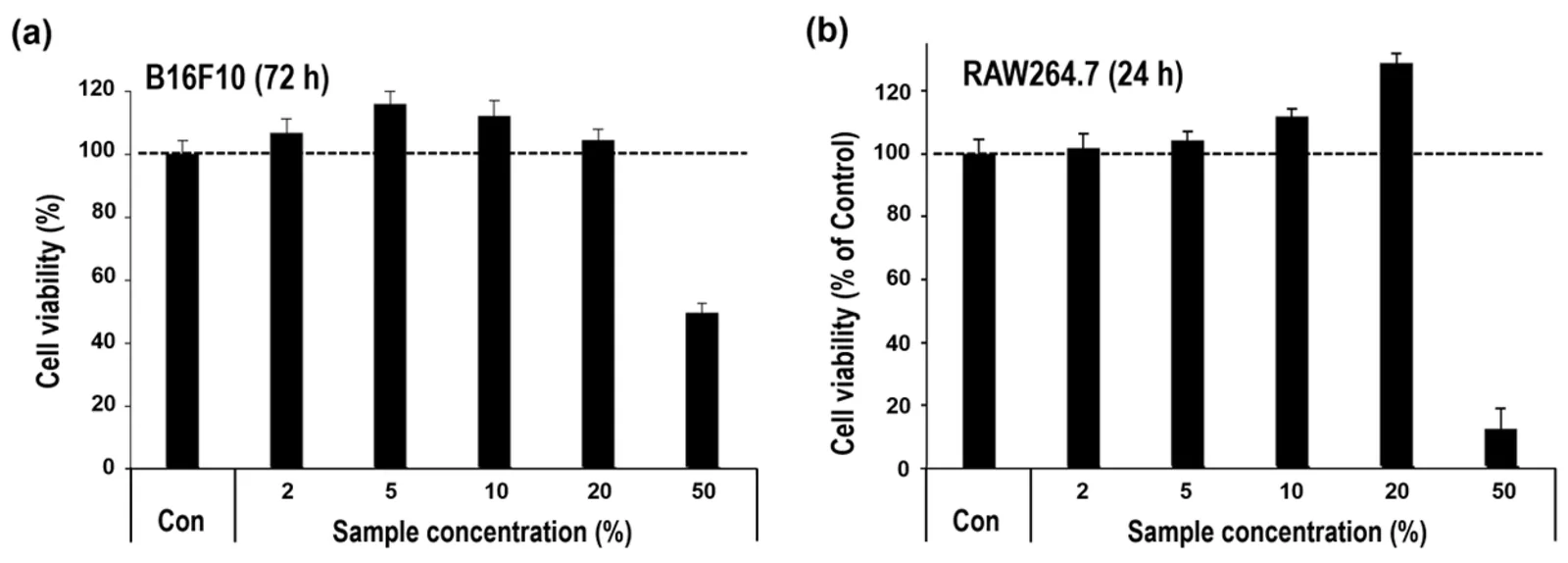
Cytotoxicity of hemp seed-derived vesicle-like nanoparticles (hs-VLNPs) in B16F10 and RAW264.7 cells. Cell viability was assessed using a CCK-8 assay after treatment with the indicated concentrations of hs-VLNPs in (**a**) B16F10 murine melanoma cells for 72 h and (**b**) RAW264.7 murine macrophages for 24 h. Cell viability is expressed as a percentage relative to the PBS-treated control. Data are presented as mean ± SD.

**Figure A3:**
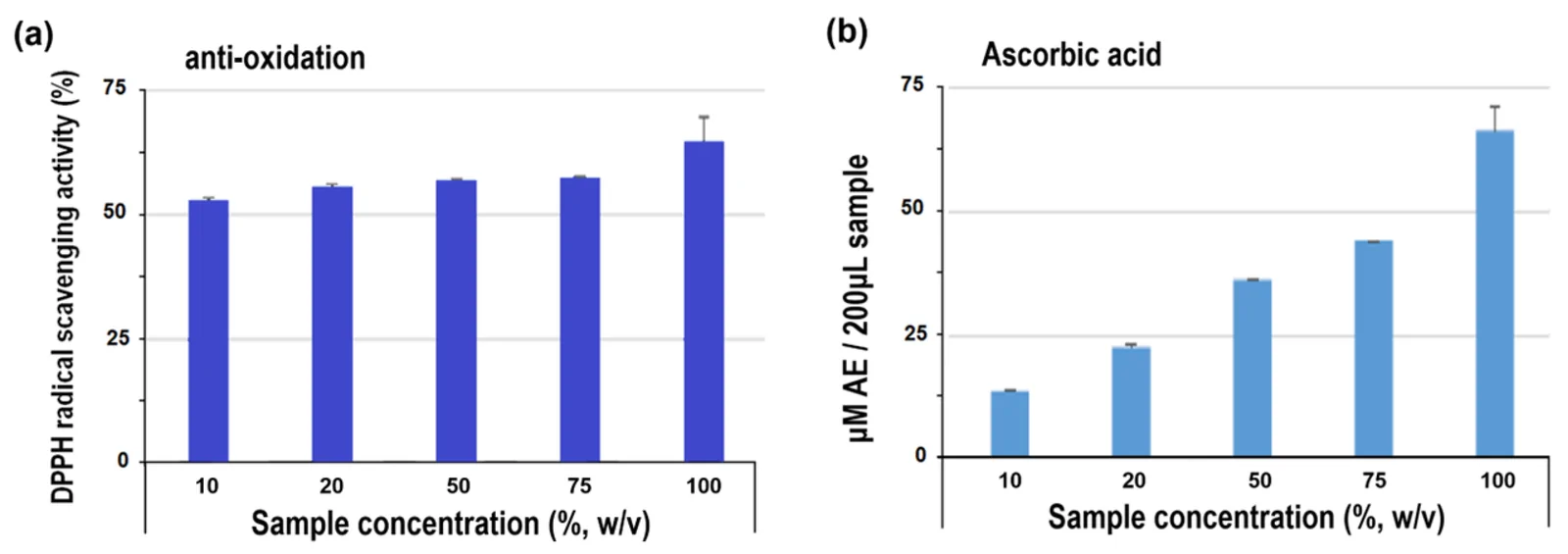
Antioxidant activity of hemp seed-derived vesicle-like nanoparticles (hs-VLNPs). (**a**) DPPH radical scavenging activity of hs-VLNPs at the indicated concentrations. (**b**) Ascorbic acid-equivalent antioxidant activity corresponding to the DPPH scavenging capacity of hs-VLNPs. Data are presented as mean ± SD.

## Data Availability

The main data supporting the results of this study are available within the manuscript and appendix. The raw data files are available for research purposes from the corresponding author upon reasonable request. Source data are provided with this paper.

## Abbreviations

EV	Extracellular vesicle
EVNP	extracellular vesicle-like nanoparticle
hs-EVNP	hemp seed-derived extracellular vesicle-like nanoparticle
HDF	human dermal fibroblast
α-MSH	alpha-melanocyte-stimulating hormone
MMP-1	matrix metalloproteinase-1

## Appendix A

**Table A1:** Primer sequences used for qRT-PCR.

Gene	Forward Primer (5′–3′)	Reverse Primer (5′–3′)
TNF-α	CTGAAAGCATGATCCGGGAC	TTAGAGAGAGGTCCCTGGGG
IL-1β	AGTGGTTTGGATGGTGCCAA	GCACCCATCATTTCCACGAGC
GAPDH	GGATTTGGTCGTATTGGGCG	ATCGCCCCACTTGATTTTGG
MMP-1	CAGAGATGAAGTCCGGTTTTTC	GGGGTATCCGTGTAGCACAT
